# 18例SMARCA4缺失或基因突变肺部肿瘤患者的临床病理与基因特征以及预后分析

**DOI:** 10.3779/j.issn.1009-3419.2026.106.09

**Published:** 2026-03-20

**Authors:** Chang LIU, Jing YANG, Fanlu MENG, Linlin ZHANG, Xin WANG, Tao YU, Diansheng ZHONG

**Affiliations:** ^1^300052 天津，天津医科大学总医院肿瘤内科; ^1^Department of Oncology, Tianjin Medical University General Hospital, Tianjin 300052, China; ^2^300052 天津，天津医科大学总医院肿病理科; ^2^Department of Pathology, Tianjin Medical University General Hospital, Tianjin 300052, China

**Keywords:** 肺肿瘤, 胸部SMARCA4缺失型未分化肿瘤, SMARCA4突变, SMARCA4 (BRG1)缺失, EGFR突变, 靶向治疗, 免疫检查点抑制剂, 预后, Lung neoplasms, Thoracic SMARCA4-deficient undifferentiated tumor, SMARCA4 mutation, SMARCA4 (BRG1) deficiency, EGFR mutation, Targeted therapy, Immune checkpoint inhibitors, Prognosis

## Abstract

**背景与目的** SMARCA4变异肺部肿瘤的诊断主要依赖于免疫组化（immunohistochemistry, IHC）检测蛋白缺失，但其与基因检测结果的一致性以及不同突变类型所导致的临床与生物学差异仍有待进一步探索。本研究旨在探讨该类肿瘤的临床病理、基因特征及预后。**方法** 回顾性纳入18例经IHC或基因检测证实为SMARCA4缺失/基因突变的肺部肿瘤连续病例，进行临床基因特征和预后分析。根据变异类型分为1类（蛋白缺失或功能丧失性基因突变，n=10）和2类（错义突变或其他意义不明突变且蛋白非缺失，n=8），并进行组间比较。**结果** 17例为非小细胞肺癌（non-small cell lung cancer, NSCLC），1例为胸部SMARCA4缺失型未分化肿瘤（SMARCA4-deficient undifferentiated tumor, SD-UT）。基因型与表型分析显示：截短突变均导致蛋白缺失，错义突变多不引起缺失，2例蛋白缺失但基因检测阴性。1类较2类变异有更高的肿瘤突变负荷（tumor mutational burden, TMB）（中位数：9.3 vs 4.7 Muts/Mb）和更低的程序性死亡-配体1（programmed cell death ligand 1, PD-L1）表达（<1%者占比：60.0% vs 25.0%）趋势，但差异无统计学意义（P>0.05）。2例表皮生长因子受体（epidermal growth factor receptor, EGFR）L858R共突变患者初始第三代EGFR靶向单药疗效不佳，联合其他治疗见效。IV期患者中1类与2类变异的中位总生存期（overall survival, OS）分别为10.3和19.9个月（P=0.967）。14例（77.8%）患者接受了免疫检查点抑制剂（immune checkpoint inhibitors, ICIs）联合化疗，其中8例（57.1%）OS超12个月，7例（50.0%）超24个月。**结论** SMARCA4基因突变与蛋白缺失不完全一致，需联合判读。SMARCA4变异与EGFR突变共存时，单药靶向疗效可能有限，应考虑联合策略。将SMARCA4变异区分为1类与2类，可能具有预后提示价值，并有助于预测肿瘤的免疫微环境状态（TMB及PD-L1）。ICIs联合化疗的方案可能是目前SMARCA4变异晚期肺部肿瘤患者的主要治疗选择之一，在本研究中显示出潜在疗效。

## Abstract (en)

**Background and objective** The diagnosis of SMARCA4-altered pulmonary tumors primarily relies on the detection of protein loss by immunohistochemistry (IHC). However, the concordance between IHC and genetic testing results, as well as the clinical and biological differences caused by various mutation types, require further investigation. This study aimed to explore the clinicopathological, genomic characteristics, as well as the prognosis, of these tumors.** Methods** A total of 18 consecutive cases of pulmonary tumor patients with SMARCA4 deficiency/mutation confirmed by IHC or genetic testing were retrospectively enrolled for clinical, genomic, and prognostic analysis. Based on the variant type, patients were categorized into Class 1 (protein loss or loss-of-function gene mutations, n=10) and Class 2 (missense mutation or other variants of unknown significance without protein loss, n=8) for intergroup comparison.** Results** Seventeen cases were diagnosed with non-small cell lung cancer (NSCLC) and one was diagnosed with thoracic SMARCA4-deficient undifferentiated tumor (SD-UT). Genotype-phenotype correlation analysis revealed that truncating mutations consistently led to protein loss, whereas missense mutations mostly did not. Two cases with protein loss but negative genetic testing results were identified. Class 1 alterations showed trends towards higher tumor mutational burden (TMB) (median 9.3 vs 4.7 Muts/Mb) and lower programmed cell death ligand 1 (PD-L1) expression (60.0% vs 25.0% with <1% expression) compared to Class 2, but the differences were not statistically significant (P>0.05). Two patients with co-occurring epidermal growth factor receptor (EGFR) L858R mutations showed poor initial response to third-generation EGFR-tyrosine kinase inhibitor monotherapy, but the efficacy was observed after combination with other therapies. Among stage IV patients, the median overall survival (OS) was 10.3 months for Class 1 and 19.9 months for Class 2 (P=0.967). Fourteen patients (77.8%) received immune checkpoint inhibitors (ICIs) combined with chemotherapy. Among them, 8 patients (57.1%) achieved an OS exceeding 12 months, and 7 patients (50.0%) exceeded 24 months.** Conclusion** SMARCA4 gene mutations are not entirely consistent with IHC protein loss, necessitating combined interpretation. When SMARCA4 alterations co-exist with EGFR mutations, targeted monotherapy efficacy may be limited, and combination strategies should be considered. Classifying SMARCA4 alterations into Class 1 and Class 2 may have prognostic implications and could help predict the tumor immune microenvironment status (TMB and PD-L1). ICIs combined with chemotherapy is currently one of the main treatment options for patients with advanced SMARCA4-altered pulmonary tumors and showed potential efficacy in this study.

SMARCA4 (BRG1)是开关/蔗糖非发酵（switch/sucrose nonfermentable, SWI/SNF）染色体重塑复合物的催化异二聚体的亚基之一^[1]^。目前SMARCA4缺失大多作为预后不良的预测指标^[2,3]^，但随着越来越多的临床前研究^[4-7]^发现，如细胞周期蛋白依赖性激酶4/6（cyclin-dependent kinase 4/6, CDK4/6）、组蛋白去甲基化酶6[lysine (K) demethylase 6, KDM6]、极光激酶A（aurora kinase A, AURKA）、共济失调毛细血管扩张和Rad3相关激酶（ataxia telangiectasia and Rad3-related kinase, ATR）抑制剂等在SMARCA4缺失型肿瘤中显示出抗肿瘤活性，其作为潜在的治疗靶点亦引起关注。多种组织来源的肿瘤均可发生SMARCA4缺失或基因突变^[8,9]^。肺部SMARCA4缺失型肿瘤可以分为两类：SMARCA4缺失型非小细胞肺癌（SMARCA4-deficient non-small cell lung cancer, SD-NSCLC）和按照2021年世界卫生组织（World Health Organization, WHO）分类命名的胸部SMARCA4缺失型未分化肿瘤（SMARCA4-deficient undifferentiated tumor, SD-UT）^[10]^，老命名方式为SMARCA4缺失型肉瘤。前者在NSCLC中的发生率约为10%^[11]^，后者更为罕见，预后更差^[12,13]^，而且，在最新版WHO分类中被更名并归类为“其他上皮源性肿瘤”。越来越多的证据^[1,14]^表明，SD-UT在组织遗传学上与癌/上皮祖细胞有关，而非原发性胸部肉瘤，甚至一些学者认为其是一种“去分化”的肺癌。也因此，SD-NSCLC与SD-UT虽被分类为不同肿瘤，但在生物学上或存在一定的渐变或过渡，在分子水平上也有一些共同之处。二者均常伴随与吸烟有关的基因组改变如丝氨酸/苏氨酸激酶11（serine/threonine kinase 11, STK11）、Kelch样ECH关联蛋白1（Kelch-like ECH-associated protein 1, KEAP1）等^[1,15]^。此外，根据WHO胸部肿瘤分类指南，SMARCA4缺陷型肿瘤的诊断通常依赖于免疫组化（immunohistochemistry, IHC）检测显示的SMARCA4蛋白完全缺失，而相应基因突变检测在诊断上并非必需^[10]^。然而，IHC结果判读存在主观性，可能遇到困难。因此，进行SMARCA4基因层面的检测亦可起到重要的辅助评估作用，且更有助于了解二者之间的一致性。再者，目前SMARCA4变异的肺部肿瘤缺乏标准方案，常规放化疗疗效有限^[12]^。鉴于上述情况，本文在探讨SMARCA4基因突变与相应蛋白表达之间关系的基础上，通过对天津医科大学总医院肿瘤内科收治的18例SMARCA4缺失或基因突变的肺部肿瘤患者的临床病理特征、基因组学信息、治疗方案及预后进行整理总结，以期阐明其生物学行为与临床特点，为判断预后和优化临床诊疗策略提供支持。

## 1 资料与方法

### 1.1 病例资料

回顾性分析2020年11月至2023年1月于天津医科大学总医院就诊的肺部恶性肿瘤患者，组织学标本基因检测提示存在SMARCA4突变；或尽管基因检测未发现其突变，但组织病理IHC染色发现SMARCA4缺失，且具备完整临床随访资料的连续病例共18例，其中13例为我院病理标本，另外5例原始标本来自外院。搜集相应的临床、病理及基因检测资料。对临床分期为IV期的患者，进一步进行生存分析，随访截止日期为2025年7月18日。资料由医院电子病例数据库及电话随访获得。本研究通过天津医科大学总医院伦理委员会批准（批件号：IRB2025-YX-653-01）。因本研究为非干预性研究，知情同意被豁免。

### 1.2 基因检测

患者的基因检测资料通过病例搜集获得，标本均来源于福尔马林固定、石蜡包埋肿瘤组织样本，基因检测结果来源于第三方商业检测机构数据，均采用二代测序（next-generation sequencing, NGS），覆盖SMARCA4基因的完整编码区及关键内含子区域，且包含肿瘤突变负荷（tumor mutational burden, TMB）。TMB的计算定义为每兆碱基中体细胞非同义突变的总数。所有样本在测序前均经过质控评估（肿瘤细胞含量评估、DNA质量评估及测序质量评估）达到合格。生信分析遵循标准流程，包括序列比对、去重、变异识别与注释。

### 1.3 病理诊断及IHC检测

对基因检测提示存在SMARCA4突变的肺部恶性肿瘤患者进行本院标本的SMARCA4 IHC染色，外院标本（共5例，占比27.8%）因回顾性分析获取困难无法进行。采用徕卡BOND-III全自动染色机进行IHC染色。SMARCA4抗体购自北京中杉金桥生物技术有限公司。按照试剂说明书标准进行结果判读。其他关注的IHC指标包括：细胞角蛋白（cytokeratin, CK）、CK7、细胞程序性死亡-配体1（programmed cell death ligand 1, PD-L1），均由本院及外院既往病理报告结果获得。

### 1.4 患者分类

根据患者的SMARCA4的IHC染色结果或基因突变类型推测蛋白的可能表达情况将患者分为两类：（1）1类变异（Class 1 alterations）：SMARCA4蛋白IHC检测缺失或基因检测提示功能丧失性变异（包括截短突变、基因融合、纯合缺失等）；（2）2类变异（Class 2 alterations）：基因检测发现SMARCA4错义突变或其他意义不明变异（variants of unknown significance, VUS），且IHC未提示蛋白缺失。对于部分特殊病例，分类遵循以下原则：若IHC提示SMARCA4蛋白缺失，即使NGS未检出突变，仍归为1类；对于错义突变相应SMARCA4蛋白表达部分弱阳性的病例，提示缺失，归为1类；根据cBioPortal数据库的分类，有记录的SMARCA4框内突变均归于VUS^[16]^，故纳入2类变异。

### 1.5 统计学分析

比较两类SMARCA4缺失或基因突变患者临床资料和肿瘤特征，根据不同资料类型，分别采用非参数检验（Mann-Whitney U检验）或Fisher确切概率法进行分析。在应用免疫检查点抑制剂（immune checkpoint inhibitors, ICIs）的晚期患者中进行无进展生存期（progression-free survival, PFS）和总生存期（overall survival, OS）的分析并在两类突变中进行比较。PFS定义为从免疫治疗开始到疾病进展或死亡的时间，OS定义为从免疫治疗开始到患者死亡的时间。未发生事件的患者在末次随访日期进行删失。采用Kaplan-Meier法进行生存分析，计算中位OS和PFS，采用Log-rank检验在两组间进行比较。应用SPSS 25.0软件进行统计学分析，统计检验采用双尾检验，P<0.05为差异有统计学意义。

## 2 结果

### 2.1 18例SMARCA4缺失或基因突变肺部肿瘤患者的临床病理基因特征

#### 2.1.1 基本临床资料

18例SMARCA4缺失或基因突变肺部肿瘤患者中，17例为NSCLC，1例根据2021年WHO胸部肿瘤分类（第5版）标准^[10]^诊断为胸部SD-UT，中位年龄为62.5岁[四分位数间距（interquartile range, IQR）：59.8，71.3]，男性占比66.7%，女性占比33.3%；吸烟者或既往吸烟者占比72.2%，不吸烟者占比27.8%；有家族肿瘤史占比55.6%，无家族肿瘤史占比44.4%；肿瘤中位最大长径为53.0（IQR: 22.0, 78.5）mm，活检类型中肺穿刺标本占比38.9%，气管镜标本占比22.2%，锁骨上淋巴结活检标本占比27.8%，手术标本占比11.1%。临床分期中可手术I-III期病例为2例（11.1%），不可手术局部晚期IIIB-IIIC期病例为4例（22.2%），IV期病例为12例（66.7%）。IV期患者中最常见转移部位为肺内转移（58.3%），其次为胸膜转移、脑转移（均占25.0%），肾上腺转移及骨转移占比均为16.7%，肝转移占比8.3%。按变异类型分类，1类变异10例，2类变异8例。1类变异中6例（60.0%）影像学表现为与胸壁关系密切的肿块影，增强扫描呈不均匀强化，8例（80.0%）存在肺门、纵隔、锁骨上淋巴结转移。SMARCA4缺失肺癌典型影像表现如图1所示。

**图1:**
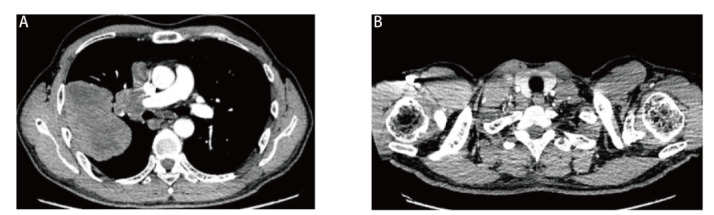
SMARCA4缺失的肺腺癌胸部强化CT表现。A：右肺上叶近胸膜肿块，最大截面积103 mm×61 mm，内部可见低密度坏死区，增强扫描呈不均匀强化，右肺门、纵隔淋巴结转移；B：双锁骨上窝淋巴结转移。

#### 2.1.2 病理类型、IHC特征

病理类型中最常见为腺癌，占比为61.1%，其次为NSCLC非特指型（not-otherwise specified, NOS），占比为16.7%，肉瘤样癌为11.1%，鳞癌及SD-UT均只有1例（5.6%）。除相应IHC结果不详的患者，SMARCA4缺失/基因突变的NSCLC均有CK或CK7的表达，SD-UT的CK表达为阴性。SMARCA4缺失型肺部肿瘤特征性病理镜下表现如图2所示。

**图2:**
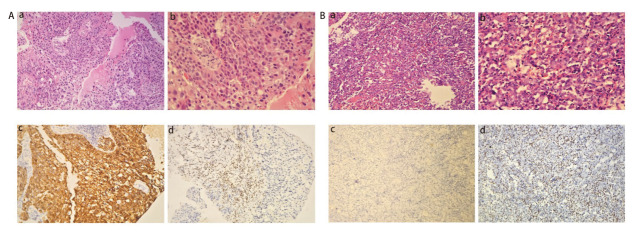
SMARCA4缺失型肺部肿瘤病理镜下表现。A：SMARCA4缺失型NSCLC，右锁骨上淋巴结标本。a：肿瘤细胞呈巢团样片状分布，可见灶性坏死（HE染色，×200）；b：肿瘤细胞稍大，胞浆丰富，嗜酸，可见核仁（HE染色，×400）；c：CK7阳性（IHC染色，×200）；d：SMARCA4阴性（IHC染色，×200）。B：胸部SMARCA4缺失型未分化肿瘤。a：肿瘤细胞呈片状分布，有灶性坏死，背景中可见大量炎性细胞浸润（HE染色，×200）；b：肿瘤细胞体积大，胞浆丰富、嗜酸，核大，核仁明显，肿瘤细胞生长活跃，核分裂易见（HE染色，×400）；c：CK阴性（IHC染色，×200）；d：SMARCA4阴性，炎性细胞作为内对照阳性表达（IHC染色，×200）。

#### 2.1.3 SMARCA4蛋白表达情况与基因突变类型关系

所有SMARCA4截短突变，除2例SMARCA4蛋白表达不详外，其余均发生SMARCA4蛋白缺失；SMARCA4剪切突变1例，SMARCA4蛋白缺失；除1例错义突变患者SMARCA4蛋白表达部分弱阳性（提示缺失）外，其余错义突变SMARCA4表达均阳性（图3）。此外，2例患者存在SMARCA4蛋白缺失，但基因检测未发现SMARCA4突变（图3），这2例患者相应病理类型均为NSCLC-NOS，且均伴肾上腺转移。

**图3:**
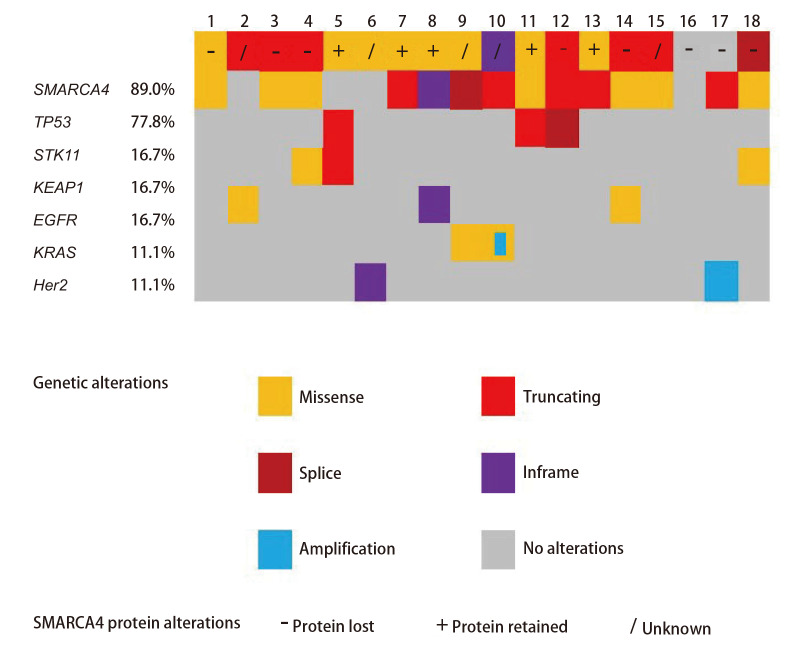
18例SMARCA4变异肺部肿瘤患者蛋白表达与共突变谱。关键共突变谱：EGFR突变3例（含21号外显子L858R突变2例，20号外显子D770_P772dup插入突变1例）；KRAS突变2例（含G12C突变合并KRAS扩增1例，G12D突变1例）；Her2突变2例（含20号外显子G778_P780dupGSP插入突变1例，Her2扩增1例）。

#### 2.1.4 基因伴随突变特征

在进行NGS基因检测的18例SMARCA4缺失或基因突变的肺部肿瘤患者中，16例（88.9%）存在SMARCA4突变（8例错义突变、6例截短突变、1例框内缺失突变、1例剪切突变），2例（11.1%）为SMARCA4野生型。SMARCA4缺失或基因突变最常伴肿瘤蛋白p53（tumor protein P53, TP53）突变（77.8%），包括7例错义突变、5例截短突变、1例框内缺失突变、1例剪切突变；与STK11共突变3例（16.7%），包括2例截短突变、1例剪切突变；与KEAP1共突变3例（16.7%），包括2例错义突变、1例截短突变；有3例（16.7%）合并表皮生长因子受体（epidermal growth factor receptor, EGFR）突变，其中2例为EGFR L858R突变，1例为EGFR 20外显子插入突变（D770_P772dup）；伴Kirsten大鼠肉瘤病毒癌基因同源物（Kirsten rat sarcoma viral oncogene homolog, KRAS）基因突变2例（11.1%），其中1例为KRAS G12D，1例为KRAS G12C合并KRAS扩增；伴人表皮生长因子受体2（human epidermal growth factor receptor 2, Her2）突变2例（11.1%），1例为20外显子插入突变（G778_P780dupGSP），另1例为Her2扩增（图3）。

#### 2.1.5 两类变异临床病理资料及PD-L1、TMB情况对比特征

18例SMARCA4缺失或基因突变肺部肿瘤患者中，1类变异10例，2类变异8例。1类患者与2类相比，中位年龄分别为60.5岁（IQR: 58.8, 71.5）和69.0岁（IQR: 62.3, 71.5），男性比例分别为80.0%（8/10）和50.0%（4/8），吸烟或既往吸烟者比例分别为80.0%（8/10）和62.5%（5/8）。两组患者的组织学类型以腺癌为主，占比分别为60.0%（6/10）和62.5%（5/8），中位TMB分别为9.3（IQR: 5.5, 13.9）和4.7（IQR: 2.1, 9.3）Muts/Mb（图4A），TMB≥10 Muts/Mb的比例分别为50.0%（5/10）和12.5%（1/8），PD-L1表达<1%的比例分别为60.0%（6/10）和25.0%（2/8）（图4B）。两类变异上述指标的组间差异均无统计学意义（P>0.05，表1）。

**图4:**
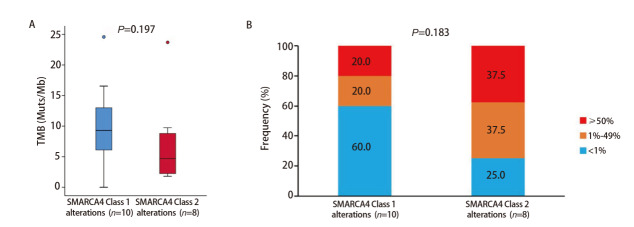
两类SMARCA4变异的中位TMB及PD-L1表达情况对比。A：中位TMB数值对比；B：PD-L1表达情况对比。

**表1:** 两类SMARCA4变异肺部肿瘤的临床病理资料及免疫治疗相关标志物（PD-L1/TMB）对比

Characteristic	SMARCA4 Class 1 alterations (n=10)	SMARCA4 Class 2 alterations (n=8)	P
Age (yr), median (IQR)	60.5 (58.8, 71.5)	69.0 (62.3, 71.5)	0.266
Sex, n (%)			0.321
Male	8 (80.0)	4 (50.0)	
Female	2 (20.0)	4 (50.0)	
Smoking status, n (%)			0.608
Never smoker	2 (20.0)	3 (37.5)	
Smoker/Former	8 (80.0)	5 (62.5)	
Histology, n (%)			0.999
Adenocarcinoma	6 (60.0)	5 (62.5)	
Other	4 (40.0)	3 (37.5)	
NSCLC-NOS	3 (30.0)	0 (0.0)	
Sarcomatoid carcinoma	0 (0.0)	2 (25.0)	
Squamous cell carcinoma	0 (0.0)	1 (12.5)	
Thoracic SD-UT	1 (10.0)	0 (0.0)	
TMB (Muts/Mb), median (IQR)	9.3 (5.5, 13.9)	4.7 (2.1, 9.3)	0.197
TMB (Muts/Mb), n (%)			0.152
<10	5 (50.0)	7 (87.5)	
≥10	5 (50.0)	1 (12.5)	
PD-L1 (TPS), n (%)			0.188
<1%	6 (60.0)	2 (25.0)	
≥1%	4 (40.0)	6 (75.0)	
PD-L1 (TPS), n (%)			0.183
<1%	6 (60.0)	2 (25.0)	
1%-49%	2 (20.0)	3 (37.5)	
≥50%	2 (20.0)	3 (37.5)	

PD-L1: programmed cell death ligand 1; TMB: tumor mutational burden; IQR: interquartile range; NOS: not otherwise specified; SD-UT: SMARCA4-deficient undifferentiated tumor; TPS: tumor proportion score.

### 2.2 SMARCA4缺失或基因突变肺部肿瘤患者的治疗及生存随访情况

#### 2.2.1 治疗及生存情况概述

18例患者的治疗模式涉及手术、放疗、化疗、ICIs、抗血管靶向、EGFR-酪氨酸激酶抑制剂（tyrosine kinase inhibitors, TKIs）、EGFR-间质上皮转化因子（mesenchymal-epithelial transition factor, MET）双抗治疗及各种治疗方式的组合，有14例（77.8%）患者接受过ICIs联合化疗，其中8例（57.1%）患者的OS超过了12个月，7例（50.0%）患者的OS超过了24个月。3例存在EGFR突变的患者均为晚期，2例合并L858R突变且SMARCA4变异类型为1类；1例合并20外显子插入突变且SMARCA4变异类型为2类。合并L858R突变的患者均接受了一线第三代EGFR-TKIs的治疗，其中1例患者2021年2月1日开始阿美替尼口服靶向治疗，3周后原发灶有所缩小，但胸水增多显著，遂行胸水引流，2021年3月5日至2021年8月19日联合贝伐珠单抗静脉输注治疗，胸水控制，靶灶缩小，评效稳定（stable disease, SD），但因出现心肌酶升高、3度皮疹，暂停原方案，更换为奥希替尼单药口服约22个月，奥希替尼口服第1年评效为SD，后续患者当地医院评估，评效具体结果不详，OS为33.4个月；另1例患者初始奥希替尼治疗后靶灶缓慢进展，联合培美曲塞+卡铂方案化疗，可见肿瘤缩小，评效达部分缓解（partial response, PR），但因不良反应耐受性差，化疗4个周期后停化疗，奥希替尼单靶继续治疗至今，评效维持PR，截至末次随访日期，OS已超过30个月，目前仍口服奥希替尼治疗中。合并20外显子插入突变的患者一线治疗方案为化疗联合ICIs，病情进展后接受过放疗、抗血管靶向联合、EGFR-MET双抗、EGFR-TKIs联合治疗，OS为24.9个月。1例患者为可手术切除的IA2期SD-UT，因病理类型差，术后接受辅助化疗，截至末次随访日期OS已达44.6个月，目前仍在随访中。

#### 2.2.2 晚期SMARCA4缺失或基因突变肺部肿瘤患者的生存情况

在上述患者中筛选出初诊时即为IV期患者共12例，其中两种类型的SMARCA4异常患者分别各占6例，进一步进行生存分析。12例患者的整体mOS为19.9个月（范围：2.4-48.3）。1类（n=6）和2类（n=6）的mOS分别为10.3（范围：2.4-48.3）和19.9[范围: 6.7-未达到（not reached, NR）]个月，差异无统计学意义（P=0.967）。

#### 2.2.3 ICIs应用与治疗效果及生存的关系

在上述晚期患者中，整个治疗期间未应用过ICIs的患者2例，应用过ICIs的患者10例，二者mOS分别为3.0和19.9个月，差异无统计学意义（P=0.457）。进一步对接受过ICIs治疗的10例患者的PFS及OS进行分析。免疫治疗阶段的mPFS为8.6个月（范围：2.4-48.3个月），10例患者整体mOS为19.9个月（范围：2.4-48.3个月），其中6例（60.0%）患者的OS超过了12个月，5例（50.0%）患者的OS超过了24个月。10例患者中1类变异4例，2类变异6例。两类患者免疫治疗阶段的mPFS分别为7.8（范围：2.4-48.3）和8.6（范围：3.4-NR）个月，差异无统计学意义（P=0.940），mOS分别为10.3（范围：2.4-48.3）和19.9（范围：5.6-NR）个月，差异无统计学意义（P=0.636）。

## 3 讨论

关于SMARCA4缺失型NSCLC及SD-UT，既往研究多对二者分别进行探讨，但考虑到二者在生物学上存在一定的连续性，且在分子水平上有共同之处，本研究将二者同时纳入。从本研究病例分布比例上，亦可看出SD-UT更为罕见。此外，对于SMARCA4缺失型NSCLC，既往研究^[11,17-19]^多侧重于病理IHC，而未涉及SMARCA4基因表达情况，或主要依据SMARCA4基因检测结果对这部分患者进行分类研究^[2,15]^。少有研究结合IHC及基因检测结果综合进行分类及分析对比。

本研究1类变异有更高的男性患者比例（80.0%）及吸烟患者比例（80.0%），这与既往文献^[20]^报道的SMARCA4缺失型NSCLC临床特征一致。SMARCA4基因突变类型与其蛋白表达缺失存在明确关联：功能丧失性突变（如截短突变）一致导致蛋白缺失；而多数错义突变则不然。此外，本研究有2例NGS检测未发现SMARCA4突变，但IHC提示SMARCA4缺失的患者。此类“基因检测阴性而蛋白表达缺失”的现象虽不常见，但已有文献^[14,21]^报道，具体原因可能与检测覆盖率低的内含子区域的结构变异（如易位）或发生了表观遗传沉默有关。这强调了在临床实践中联合使用IHC与NGS进行综合判读的重要性。本研究发生SMARCA4蛋白缺失但NGS未检出突变的2例患者，组织学均表现为分化差，因缺乏明确分化特征而暂归类为NSCLC-NOS。国内相关指南与共识^[22]^提示，对分化差的癌或恶性肿瘤可行SMARCA4 IHC。这也是本研究对上述2例NSCLC-NOS病例主动加染SMARCA4的依据。亦有学者重点关注了病理分型为NSCLC-NOS的SMARCA4表达情况，纳入了110例NSCLC-NOS患者，均接受SMARCA4 IHC染色，发现其中有9例（8.2%）为SMARCA4缺失型^[11]^。因此，无论基因检测是否提示发生SMARCA4突变，当发现肺部肿瘤患者病理类型分化差时，进行SMARCA4 IHC染色可能是必要的。此外，本研究的这2例患者恰巧都存在肾上腺转移。在1篇纳入21例SMARCA4缺失的胸部肉瘤（2021年后更名为“SD-UT”）的文献报道^[23]^中，肾上腺转移占比最高（47.6%），其次为肺转移（28.6%）和骨转移（23.8%）。另1篇纳入20例IHC证实为SMARCA4缺失型肺腺癌患者的文献^[21]^中，肾上腺转移在晚期患者中的占比达38.5%。近期亦有文献^[3]^发现，IHC提示SD-NSCLC患者肾上腺转移的比例显著高于SMARCA4蛋白保留型患者（15% vs 7%, P=0.020）。发生肾上腺转移的NSCLC患者预后差^[24]^，而SMARCA4缺失为何与肾上腺转移存在一定关系，有待进一步研究。

回顾既往文献，无论SMARCA4变异的NSCLC抑或是SD-UT，通常有更高的TMB，但PD-L1常为低表达或阴性，最常伴随TP53共突变，其次为KEAP1、STK11及KRAS共突变，与目前主要的驱动基因EGFR、间变性淋巴瘤激酶（anaplastic lymphoma kinase, ALK）、ROS原癌基因1（ROS proto-oncogene 1, ROS1）、MET、转染重排原癌基因（rearranged during transfection proto-oncogene, RET）突变等存在互斥性，其中与EGFR突变的互斥性最强^[2,12,14,25 -27]^。本研究患者合并常见基因共突变的情况与既往文献报道一致，1类变异较2类变异有更高的TMB及PD-L1阴性患者比例。但合并EGFR突变的比例高于文献报道，且更少见的是，2例EGFR L858R突变患者合并SMARCA4变异类型均为1类。这2例患者初始第三代EGFR-TKIs单药治疗均效果不佳，1例胸水显著增多，另1例靶灶缓慢进展，联合抗血管靶向/化疗治疗后可见一定疗效。这2例虽因不良反应不耐受停止了联合治疗，减为第三代EGFR-TKIs单药，但或因前期联合治疗后，肿瘤负荷降低，原耐药前体细胞减少，后续奥希替尼单靶维持治疗，原治疗效果巩固，1例维持用药22个月，另1例截至末次随访日期，疗效仍维持PR。目前有关SD-NSCLC合并EGFR敏感突变的报道较少，尚未检索到伴EGFR 19外显子缺失突变相关治疗的病例报道，有零星合并EGFR L858R突变的报道，单药靶向治疗效果欠佳，预后差^[3,19]^。以上提示在SMARCA4异常背景下，即使存在经典EGFR驱动基因突变，也需审慎评估单纯EGFR-TKIs的治疗效能，未来或应探索靶向联合治疗等更积极的策略。但亦有1例SD-UT伴ALK融合突变的个案^[28]^，一线阿来替尼单药靶向治疗，病灶显著完全缓解。可见伴可靶向驱动基因突变的SMARCA4缺失型肺部肿瘤，靶向治疗依然是基础，而对于与SMARCA4突变互斥性最强的EGFR突变来说，本研究发现靶向联合其他治疗及重点病灶的局部处理或可对预后改善有所帮助。

对于晚期NSCLC患者来说，文献报道SMARCA4 1类突变预后最差，2类突变介于野生型和1类突变之间^[2]^，2类突变多为错义或VUS，其本身对SMARCA4蛋白ATP酶功能或染色质重塑复合物组装的影响较弱或不确定^[29]^。因此，这类肿瘤可能保留了部分野生型功能，其生物学行为可能介于完全缺失（1类）与野生型之间，也被认为存在有害性^[3]^，这也体现了基因检测对IHC的补充。1类突变患者的mOS不同文献报道在4.4-12.2个月^[2,3,26]^，与文献报道类似，本研究1类变异晚期患者mOS为10.3个月，2类变异mOS为19.9个月，差异虽然无统计学意义，也体现了1类变异预后差于2类的趋势。此外，因SMARCA4变异型肺部肿瘤不常伴可靶向的驱动基因突变，且对常规细胞毒性化疗反应不佳，ICIs的加入在该类患者中被给予更多期望^[30]^。一项大型的回顾性研究^[2]^显示，SMARCA4突变的晚期NSCLC，接受过ICIs治疗比未接受ICIs的患者有更好的OS，且1类突变对于免疫治疗的客观缓解率（objective response rate, ORR）好于2类突变及SMARCA4野生型，遗憾的是，这种ORR的优势并未转换成PFS及OS的获益。另一项回顾性研究^[3]^显示，SMARCA4缺失型NSCLC，接受免疫联合化疗的治疗方式较其他方式患者有显著的OS获益。本研究18例SMARCA4缺失/基因突变患者的治疗模式更为综合，有14例接受过免疫联合化疗的治疗模式，其中一半以上的患者OS已超过1年。此外，本研究初步发现不同类别的SMARCA4变异与差异化的肿瘤免疫微环境（如TMB、PD-L1表达）有关联的趋势，这或许能为未来筛选更可能从免疫治疗中获益的亚群提供生物学依据。本研究数据进一步支持ICIs联合化疗可能是SMARCA4变异肺部肿瘤当前可行的主要选择。

本研究存在一些局限性：（1）本研究仅纳入18例患者，样本量较小，这限制了研究结果的统计效能和外部推广性，且为单中心回顾性分析，可能带来选择偏倚与混杂因素控制不足的问题，为保持数据的完整性与代表性，纳入了研究期间内所有符合条件的连续病例，未进一步增加排除条件，以避免因过度筛选而损失本已有限的样本量，尽可能反映真实世界情况；（2）本研究有5例（27.8%）患者的原始病理标本来源于外院，但患者治疗和随访均在同一中心，不破坏队列连续性。受回顾性研究条件所限，无法对该5例标本补充进行SMARCA4蛋白的IHC检测，为尽可能减少由此产生的潜在信息偏倚，依据其SMARCA4基因检测结果，参照既往文献及cBioPortal数据库的证据，将5例患者进行分类，通过基因型进行的合理分类已最大程度地弥补了这一信息缺口，因此其对研究主要结论产生的偏倚可能有限。未来尚需通过更大样本量的多中心研究及前瞻性设计获取标准化标本来进一步验证本研究的初步发现。

通过本次研究观察到SMARCA4基因突变与其编码蛋白的缺失并非完全一致，这提示在临床与科研中，需将“SMARCA4基因突变”与“SMARCA4蛋白缺失”作为相关但独立的概念进行区分与联合解读，二者具有重要的互补价值。此外，对于一些分化差的肺部肿瘤，或者NSCLC-NOS，临床医生应积极考虑与病理科沟通，增加SMARCA4 IHC检测。同时也更需了解SMARCA4缺失或基因突变患者的伴随突变情况，尽管SMARCA4突变与EGFR突变存在互斥性，但本研究中发现了少数共突变病例，且在这些患者中观察到单纯靶向治疗疗效有限的现象，靶向联合及借助其他综合治疗手段或可改善预后。同时，本研究中多数晚期患者接受了免疫联合化疗，与单纯化疗相比，联合免疫的方案显示出了潜在的生存获益趋势。此外，数据提示不同类型的SMARCA4变异（1类与2类）可能在TMB及PD-L1表达上存在差异。综上，本研究的价值在于，将SMARCA4变异肺部肿瘤“缺乏有效方案”这一临床困境，转化为若干可供验证的具体方向：对于合并驱动基因共突变患者，应探索超越单药靶向的综合策略；对于广泛群体，免疫联合化疗值得作为优先考量，同时应深入开展生物标志物研究以优化患者选择。需要强调的是，以上结论均源于本回顾性研究的有限样本。其核心价值在于为未来研究提供初步线索与假设。特别是治疗相关发现，亟需在前瞻性、大样本研究中进一步验证，以明确其临床指导意义。
